# Vitamin A supplementation in Tanzania: the impact of a change in programmatic delivery strategy on coverage

**DOI:** 10.1186/1472-6963-6-142

**Published:** 2006-11-01

**Authors:** Honorati Masanja, Joanna Armstrong Schellenberg, Hassan M Mshinda, Meera Shekar, Joseph KL Mugyabuso, Godwin D Ndossi, Don de Savigny

**Affiliations:** 1 Ifakara Health Research & Development Center, Ifakara, Morogoro, Tanzania; 2 Department of Epidemiology, Swiss Tropical Institute, Basel, Switzerland; 3 Department of Infectious and Tropical Disease, London School of Hygiene and Tropical Medicine, Keppel Street, London, UK; 4 Human Development Network (HDNHE), The World Bank, 1818 H Street, NW, Washington DC 20433, USA; 5 Helen Keller International, Dar es Salaam, Tanzania; 6 Tanzania Food and Nutrition Center, Dar es Salaam, Tanzania; 7 Tanzania Essential Health Interventions Project, Dar es Salaam, Tanzania

## Abstract

**Background:**

Efficient delivery strategies for health interventions are essential for high and sustainable coverage. We report impact of a change in programmatic delivery strategy from routine delivery through the Expanded Programme on Immunization (EPI+) approach to twice-yearly mass distribution campaigns on coverage of vitamin A supplementation in Tanzania

**Methods:**

We investigated disparities in age, sex, socio-economic status, nutritional status and maternal education within vitamin A coverage in children between 1 and 2 years of age from two independent household level child health surveys conducted (1) during a continuous universal targeting scheme based on routine EPI contacts for children aged 9, 15 and 21 months (1999); and (2) three years later after the introduction of twice-yearly vitamin A supplementation campaigns for children aged 6 months to 5 years, a 6-monthly universal targeting scheme (2002). A representative cluster sample of approximately 2,400 rural households was obtained from Rufiji, Morogoro Rural, Kilombero and Ulanga districts. A modular questionnaire about the health of all children under the age of five was administered to consenting heads of households and caretakers of children. Information on the use of child health interventions including vitamin A was asked.

**Results:**

Coverage of vitamin A supplementation among 1–2 year old children increased from 13% [95% CI 10–18%] in 1999 to 76% [95%CI 72–81%] in 2002. In 2002 knowledge of two or more child health danger signs was negatively associated with vitamin A supplementation coverage (80% versus 70%) (p = 0.04). Nevertheless, we did not find any disparities in coverage of vitamin A by district, gender, socio-economic status and DPT vaccinations.

**Conclusion:**

Change in programmatic delivery of vitamin A supplementation was associated with a major improvement in coverage in Tanzania that was been sustained by repeated campaigns for at least three years. There is a need to monitor the effect of such campaigns on the routine health system and on equity of coverage. Documentation of vitamin A supplementation campaign contacts on routine maternal and child health cards would be a simple step to facilitate this monitoring.

## Background

Vitamin A supplementation is one of the best proven, safest and most cost-effective interventions in public health [1]. In populations where vitamin A deficiency is of public health importance [2], vitamin A supplements are recommended as prophylaxis and as treatment for at-risk groups and sick individuals respectively. Approximately two-thirds [3] of the 10.8 million child deaths [4] that presently occur can be prevented by available interventions of which vitamin A supplementation (VAS) is one. A meta-analysis of several large vitamin A trials has shown that improving vitamin A status reduces mortality rates between 23–34% among children six months to five years of age if vitamin A supplementation is given at least twice per year at coverage rates of at least eighty percent [5,6].

In Tanzania, efforts to combat vitamin A Deficiency (VAD) started through a disease-targeted approach in 1987. This was confined to government-owned primary health care facilities and targeted therapeutically to children between 6–59 months suffering from xerophthalmia or diseases precipitating VAD. Consequently, many young children at risk of VAD in Tanzanian communities were not treated with vitamin A. In 1997, Vitamin A supplementation was introduced through the routine Essential Drugs Programme (EDP) for post partum mothers' and children at 9 months together with measles vaccination.

During measles vaccine campaigns in late 1999 and 2000, children between 6–59 months from 30 and 52 districts respectively of mainland Tanzania also received vitamin A supplements. Post campaign vitamin A supplementation coverage levels in these pilot campaigns were estimated at 94% and 99% in 1999 and 2000 respectively (Mugyabuso JKL. paper presented at the Annual EPI evaluation meeting 2002). Based on these results and similar lessons from the Philippines [7], UNICEF initiated and supported the national bi-annual implementation of VAS in children 6 months to 5 years in Tanzania starting in 2001. Advocacy, social mobilization and campaigns through national mass media agencies were used to create awareness in communities. The estimated coverage for targeted children in June and December 2001 from routine statistics was 80% and 91% respectively (Mugyabuso JKL, paper presented at the Annual EPI evaluation meeting 2001).

Here we describe the effect of the change in programmatic delivery strategy of vitamin A supplementation on coverage as documented through two independent household-level child health surveys carried out prior to the introduction of the twice-yearly mass distribution strategy (1999) and three years later (2002). We also describe factors associated with access to the intervention.

## Methods

This study was part of a larger evaluation of the effectiveness and cost of health system and facility-based Integrated Management of Childhood Illness (IMCI). Details of the study area have been described elsewhere [8]. In brief, Rufiji and Kilombero districts are low-lying and much of the land has fertile alluvial soil of the Rufiji and Kilombero River plains respectively. Morogoro Rural and Ulanga are mountainous with some low-lying areas. The main economic activities of the population are subsistence farming and fishing. The four districts have an estimated total population of about 1.2 m people [9]. Morogoro Rural district participated in the measles vaccine campaign, which also included pilot vitamin A supplementation in the late 1999 but not in 2000. Kilombero district participated in the measles campaign with pilot vitamin A supplementation in 2000. Rufiji and Ulanga districts were not part of either pilot campaign.

A representative cluster sample of approximately 2,400 rural households was obtained from the four districts in July–August 1999 and 2002. In 1999, 30 clusters were selected from each of three districts and 35 clusters from Kilombero District. Villages (clusters) were selected with probability proportional to size, and one kitongoji (sub-village with approximately 100 households) was selected at random from each selected village. 20 households were selected using a modified EPI-type scheme [10] that guaranteed an equal probability of selection for every household. In 2002, the same villages were selected but the sampling of sub-villages and households was repeated with replacement. The chances of visiting the same households were small. Ifakara town in Kilombero district is the largest urban area in the survey: ten additional clusters from this town have been omitted from the analysis described here, so that all results refer to rural areas.

A structured questionnaire about the health of all children under the age of five years was administered to consenting heads of the households. We documented background information of all children in the household, information on proxy indicators of household socio-economic status such as household ownership of a radio, a tin roof, a bicycle, educational level and occupation of the head of household. Mothers or caretakers of children were interviewed about their educational level and knowledge of child health danger signs (fast breathing, difficult breathing, fits or convulsions, very sleepy, vomiting all ingested material or inability to drink or breastfeed). Vaccination history was recorded from health cards or mother's record books for those who had one. Where no could be found, mothers or caretakers were asked whether the child had DPT immunizations and the number of doses received. Mothers or caretakers were shown a vitamin A capsule and were asked whether their children had ever received a similar one, and if so, the month and year of the most recent dose.

The study received ethical clearance from the institutional review board of the Ifakara Health Research and Development Centre and the national Tanzanian Medical Research Coordinating Committee.

### Data analysis

Data were double-entered and verified using FoxPro (version 2.6, Microsoft Corporation, Seattle, WA, USA) at Ifakara Health Research and Development Centre (IHRDC). Range checks and internal consistency were performed before analysis in Stata (version 8.2, Stata Corporation, College Station, TX, USA).

Since villages (clusters) were our unit of sampling, individual analysis methods were not appropriate due to artificially small standard errors. Hence we employed cluster-sampling analysis methods in STATA to obtain correct standard errors and confidence intervals for our parameter estimates [11-13]. We used standard STATA svy*tab *command to produce design-based F-tests. Vitamin A supplementation coverage was defined as the number of children between 1 and 2 years of age receiving vitamin A in the six months prior to the date of the survey divided by the total number of children between 1 and 2 years in the survey [14]. A relative index of socio-economic status was constructed by combining documentation of household characteristics (type of roof), assets (house ownership, bicycle, net, animals, and fowls), income sources (income of head apart from farming, maternal income) and education (head and maternal education). We used principal components analysis to estimate the appropriate weights or "scores" for the index [15]. Quintiles of children were developed based on their household socio-economic score.

## Results

A total of 385 and 388 children aged between 1 and 2 years who had complete data on vitamin A supplementation were sampled from 2,131 and 2,027 households in 1999 and 2002 surveys respectively. Table 1 shows selected background of the study subjects from the two surveys. Approximately 1% (1999) and 0.4% (2002) of the households refused to participate in the surveys.

Overall coverage of vitamin A increased from 13.2% [95% CI: 9.8–17.7%] in 1999 to 76.3% [95% CI: 71.5–80.5%] (Table 2). As expected, maternal education was associated with VAS coverage, although the difference reached conventional statistical significance only in 1999 (p = 0.02), and not in 2002 (p = 0.08). Coverage of VAS was similar in the two surveys for both boys and girls and did not vary by vaccination status. However, in the 2002 survey, maternal knowledge on child health danger signs was found to be negatively associated with VAS coverage, which was about 10 percentage points lower in mothers who knew at least two danger signs than in those who did not. The VAS achieved a major increase in coverage without any decrease in socio-economic equity: coverage was uniformly distributed by socio-economic status before and after the campaigns, as well as across the districts.

Multivariate analysis of the association between maternal education, knowledge of danger signs and vitamin A supplementation gave similar conclusions (data not shown).

## Discussion

This study has revealed that a change in the delivery strategy of interventions in a health system can have important effects on coverage without compromising equity. Our data has shown that vitamin A supplementation coverage in children between 1 and 2 years of age increased from 13% in 1999 to 76% in 2002. National coverage figures from the 1999 Tanzania Reproductive and Child Heath Survey (TRCHS) were similar (14%) [16] to what we observed in study districts. However more recent national coverage estimates from the 2004/2005 DHS, which includes around 7,000 children [17], are rather lower at (46%), than we found in our 2002 survey. These differences could be due to geographic variations in coverage or to a drop between 2002 and 2004. In contrast, 85% and 90% coverage at national level have been recently reported by two other sources (Ndossi, G.D. 2004 and Helen Keller International, 2004), the second of which was a population based assessment in 12,000 children from 21 regions of mainland Tanzania. The most likely explanation for the discrepancies between the 2004/05 DHS and HKI surveys, both of which cover the entire country, are a combination of survey timing and differences in questionnaires wording and implementation. The HKI survey was conducted during August 2004, and had a short recall period, as it was little more than a month after the June 2004 VAS campaign. The DHS was conducted from October 2004 to January 2005. Further, the HKI survey question on VAS had a specific point reference of June 2004 whereas the DHS question referred to a 6 month period prior to the survey (giving the scope to capture VAS provided through EPI+ as well as disease targeted approaches, which would have increased coverage estimates). Interview techniques, whether or not mothers were actually shown the capsules, and whether the capsules shown were of the right colour, could also help to explain the differences observed (Mugyabuso, report in preparation). Sampling methodology also differed between the surveys.

We found no evidence of socio-economic inequities in vitamin A supplementation in either 1999 or 2002, despite the major increase in coverage, unlike what has been reported elsewhere [18]. By socio-economic inequity in VAS we mean the trend in coverage level across socio-economic quintiles. New health interventions are often embraced by the richest first and followed gradually by the poorest [19]. So that one would expect equity to get worse as coverage increases before it can get better. Here we found no evidence of increased inequity despite a major increase in coverage and this highlights the need for devising ways to maintain these achievements [20]. We did not find any evidence of gender or district differentials in VAS coverage. Our results on the lack evidence of inequity in gender are similar to what was reported by Bishai and colleagues [21]. A negative association between vitamin A supplementation and maternal knowledge of child health danger signs was rather unexpected and think it is likely to be a chance finding than a real difference.

Vitamin A supplementation campaigns in Tanzania have been concerted efforts by the Government and stakeholders from the health sector. To maximize coverage, activities were strategically integrated into commemoration of the Day of the African Child and World AIDS Day events. During implementation, partnerships were forged between the Presidents Office for Regional Administration and Local Government (PORALG), Ministry of Health and the Tanzania Food and Nutrition Centre (TFNC). Funding support came primarily from UNICEF and vitamin A supplies from Canadian International Development Agency (CIDA). Other agencies and Non Governmental Organizations such as USAID and Plan International also joined these efforts, albeit in later rounds.

This study had two potential limitations. First, we depended largely on the mothers' or caretakers' accounts of the vitamin A supplementation status of their children. We also checked the child's MCH card or notebook for information about vitamin A supplementation but this had limited use as the campaign staff were trained not to record the vitamin A dose on the children's health cards. Secondly, neither the 1999 nor the 2002 household surveys were designed solely to evaluate the change in delivery strategy or policy, but were rather designed to measure coverage of various interventions for children under five, vitamin A supplementation being one, as recommended by UNICEF and WHO [14]. Thirdly, the increase in vitamin A supplementation coverage may have been due in part to other factors and not to the change in the delivery strategy above. However, we are not aware of any factors that could be responsible for the dramatic increase in coverage despite a comprehensive investigation of contextual factors [22,23]

Although the bi-annual campaign-based strategy for vitamin A delivery resulted in a major increase in coverage, it remains important that this approach sustains universal coverage while not undermining routine health programs [24].

One among the many obstacles for health systems in low-income countries is fragmented health information systems. Reliable and timely health information is an essential foundation for public health action. Such systems are vital for informing decision-makers to enable them to identify problems and needs, track progress, evaluate the impact of interventions, and make evidence-based decisions on health policy and effect policy change. Routine health programs may be weak due to poorly motivated and often overburdened health staffs, whose job includes reporting from multiple data collection systems and who suffer and who suffer from a lack of supervision. The VAS coverage rates that we have observed so far in Tanzania need to be sustained and monitored regularly if we are to reach the MDGs in 2015. The MCH "Road to Health" cards offer a practical opportunity to capture information on interventions that children actually receive through routine systems or campaigns.

During the mass distribution campaigns, vitamin A supplementation was not recorded on the health card. If campaigns are going to continue, monitoring of progress would be greatly simplified if planners ensure that information for each individual child is recorded. To our knowledge, vaccines or vitamin A supplementation campaigns that are outside routine EPI are not generally registered on children's health cards.

Coverage figures of the campaigns since 2001 have been recorded on tally sheets and subsequently summary sheets that were later collated by Tanzania Food and Nutrition Centre. The problem with this approach is the potential to over-estimate coverage because of lack of proper denominators, or inflated numerators due to multiple doses to the same children. Documenting any extra contacts such as National Immunization Days (NID) or other related health events on health cards not only offers a useful opportunity to reliably estimate coverage figures, but also creates a less demanding and more integrated health information system. In our study, we used information provided by mothers as well as that written on health cards to estimate coverage figures. This source of information is also open to error but is likely to be a conservative estimate, and has the advantage of being household-based [25].

Social mobilization campaigns are good for catch-up coverage over a short period of time. This coverage has been sustained for at least three years. In spite of the campaigns, national VAS coverage may have dropped as low as 46% in 2004 [17]. Given the evidence on the cost per death averted in Tanzania [26] and the role vitamin A can play in reducing child mortality, allocation of resources required to implement such child health interventions needs to be maintained.

## Conclusion

We conclude that, since routine services at present are not geared towards monitoring vitamin A supplementation campaign contacts, deliberate efforts must be taken to enable capturing of such information at both routine contacts and campaigns. This will facilitate the monitoring of the effect of campaigns on routine health systems, equity of coverage and tracking progress on child survival.

## Abbreviations

EPI Expanded Programme on Immunization

DPT Diphtheria Pertussis, Tetanus

VAS Vitamin A Supplementation

VAD Vitamin A Deficiency

UNICEF United Nations Children Education Fund

IMCI Integrated Management of Childhood Illnesses

IHRDC Ifakara Health Research and Development Center

TRCHS Tanzania Reproductive and Child Health Survey

TFNC Tanzania Food and Nutrition Center

PORALG Presidents Office for Regional Administration and Local Government

AIDS Acquired Immune Deficiency Syndrome

CIDA Canadian International Development Agency

USAID United States Agency for International Development

MCH Maternal and Child Health

WHO World Health Organization

NID National Immunization Day

## Competing interests

The author(s) declare that they have no competing interests.

## Authors' contributions

HM conceived the idea and participated in the design of the study, conducted the analysis and writing the manuscript. JAS participated in conceiving the idea, study design, coordination of the study and writing the manuscript. DdS participated in the design of the study and writing the manuscript. HMM participated in the coordination of the study and writing the manuscript. MS, JKLM and GDN participated in writing the manuscript. All authors read and approved the manuscript.

## Pre-publication history

The pre-publication history for this paper can be accessed here:



## Figures and Tables

**Table 1:** Age, sex, maternal education and socioeconomic status of study population in 1999 and 2002 surveys

Characteristics	Survey year			
	
	1999		2002	
	
	n	%	n	%
Sex (N = 385 in 1999, 388 in 2002)				
Female	197	51.2	183	47.2
Male	188	48.8	205	52.8
Age distribution‡				
6–11 months	467	25.2	401	21.3
1–2 years	385	20.7	388	20.6
2–3 years	353	19.0	406	21.6
3–4 years	348	18.7	356	19.0
4–5 years	304	16.4	330	17.5
DPT vaccination (N = 385 in 1999, 387 in 2002)				
Not vaccinated	105	27.3	91	23.5
Vaccinated	280	72.7	296	76.5
Mothers education (N = 385 in 1999, 388 in 2002)				
None	129	33.5	100	25.7
Primary	243	63.1	280	72.2
Secondary/Higher	13	3.4	8	2.1
Knowledge of ≥2 danger signs (N = 353 in 1999, 375 in 2002)				
No	281	79.9	244	65.1
Yes	71	20.1	131	34.9
Wealth quintiles (N = 350 in 1999, 376 in 2002)				
Poorest	60	17.1	73	19.4
Second poorest	56	16.0	77	20.5
Middle	80	22.9	70	18.6
Second richest	73	20.9	82	21.8
Richest	81	23.1	74	19.7
District (N = 385 in 1999, 388 in 2002)				
Morogoro	79	20.5	95	24.5
Rufiji	99	25.7	108	27.8
Ulanga	111	28.8	114	29.4
Kilombero	96	25.0	71	18.3

^‡ ^All children under five year included here

**Table 2:** Vitamin A coverage in children (12–23 months) by selected characteristics in 1999 and 2002

Characteristics	1999 Survey					2002 Survey				
	
	n	N	%	95%CI	p value^a^	n	N	%	95%CI	p value^a^
VAS coverage										
All districts	51	385	13.2	9.8–17.7		296	388	76.3	71.5–80.5	
Sex										
Male	27	188	14.4	9.8–20.5	0.53	161	205	78.5	71.8–93.9	0.28
Female	24	197	12.2	7.9–18.4		135	183	73.8	66.8–79.7	
DPT vaccination										
Vaccinated	37	280	13.2	9.5–18.1	0.97	229	296	77.4	71.6–82.3	0.34
Not vaccinated	14	105	13.3	7.6–22.3		66	91	72.5	63.2–80.2	
Maternal education										
No	10	129	7.8	4.2–13.8	0.02	70	100	70.0	60.2–78.3	0.08
Primary	37	243	15.2	11.2–20.3		218	280	77.9	72.4–82.5	
Secondary	4	13	30.8	12.0–59.1		8	8	100.0	-	
Knowledge of ≥2 danger signs										
No	35	282	12.4	9.1–16.8	0.09	195	244	79.9	74.4–84.5	0.04
Yes	14	71	19.4	11.5–31.8		91	131	69.5	60.0–77.6	
Socio-economic status										
Poorest	5	60	8.3	3.7–17.8	0.64^b^	56	73	76.7	67.5–84.0	0.68^b^
Very Poor	10	56	17.9	9.6–30.9		59	77	76.6	65.5–85.0	
Poor	9	80	11.3	5.7–21.1		49	70	70.0	56.7–80.6	
Less Poor	10	73	13.7	6.7–26.0		65	82	79.3	68.7–86.9	
Least Poor	12	81	14.8	8.1–25.5		58	74	78.4	67.6–86.3	
District										
Morogoro	12	79	15.2	8.2–26.5	0.85	73	95	76.8	66.9–84.5	0.94
Rufiji	13	99	13.1	6.6–24.3		81	108	75.0	65.3–82.7	
Ulanga	16	111	14.4	8.4–23.6		89	114	78.1	69.3–84.9	
Kilombero	10	96	10.4	5.5–18.9		53	71	74.6	61.6–84.4	

^a ^Pearson chi-squared statistics corrected for survey design

^b ^P-level for F-test for linear trend in proportions, using logistic regression adjusting for clustering
